# Heterozygous expression of myocilin glaucoma mutants increases secretion of the mutant forms and reduces extracellular processed myocilin

**Published:** 2008-11-21

**Authors:** José-Daniel Aroca-Aguilar, Francisco Sánchez-Sánchez, Francisco Martínez-Redondo, Miguel Coca-Prados, Julio Escribano

**Affiliations:** 1 Área de Genética, Facultad de Medicina/Centro Regional de Investigaciones Biomédicas (CRIB), Universidad de Castilla-La Mancha, Albacete, Spain; 2 Cooperative Research Network on Age-Related Ocular Pathology, Visual and Life Quality, Instituto de Salud Carlos III, Madrid, Spain; 3 Department of Ophthalmology and Visual Science, Yale University School of Medicine, New Haven, CT

## Abstract

**Purpose:**

Heterozygous mutations in the myocilin gene (*MYOC)* cause glaucoma by an unknown mechanism. *MYOC* encodes an extracellular protein of unidentified function that undergoes intracellular endoproteolytic processing in the secretory pathway. It has been described that co-expression of wild-type/mutant myocilin reduces the secretion of the wild-type protein and that single expression of glaucoma myocilin mutants reduces its proteolytic processing. However, the effect of wild-type myocilin on mutant myocilin secretion and how mutant myocilin affects the proteolytic processing of wild-type myocilin have not been investigated. We herein analyze these two issues.

**Methods:**

We modeled the heterozygous state for 4 missense (E323K, R346T, P370L, D380A) and 1 nonsense (Q368X) myocilin mutants by transiently co-expressing each mutant with the wild-type protein in HEK-293T cells. Recombinant mutant and wild-type myocilin in both culture media and cellular fractions were quantified by western immunoblot and densitometry.

**Results:**

A 24 h transient co-expression of each myocilin mutant with the wild-type protein elicited an augmented secretion of the mutant forms from 1.5 fold (D380A) to 5.4 fold (E323K). Under such conditions, extracellular mutant myocilin represented up to 20% of the total mutant protein. Other than this effect, secreted wild-type myocilin significantly decreased from 2.6 fold (E323K) to 36 fold (Q368X). When myocilin proteolytic processing was enhanced (96 hour co-expression) the extracellular amount of wild-type processed myocilin diminished from approximately 2.1 fold (E323K) to 6.3 fold (P370L). Nonreducing SDS-PAGE indicated that extracellular myocilin resulting from 24 h co-expression of wild-type myocilin and each of the 4 missense mutants forms hetero-oligomers and that glaucoma mutations do not increase the size of myocilin aggregates.

**Conclusions:**

Increased extracellular levels of mutant myocilin expressed in heterozygosis may play a relevant role in glaucoma pathogenesis. This effect is likely the result of intracellular mutant/wild-type myocilin hetero-oligomerization.

## INTRODUCTION

Glaucoma encompasses a heterogeneous group of neurodegenerative diseases as a result of the progressive degeneration of the optic nerve and loss of visual fields. Primary open-angle glaucoma (POAG; OMIM 137760) is the most frequent type of glaucoma. This disease is the second leading cause of bilateral blindness in developed countries. Indeed, it is estimated that 3-5% of the world population over 40 years of age will develop glaucoma [1], affecting some 60 million people by the year 2010 [2]. Elevated intraocular pressure (IOP) is the main known risk factor of this disease. In most POAG patients increased resistance to the outflow of aqueous humor (AH) in the trabecular meshwork (TM) results in an increment of IOP, causing ganglion cell death in the neural retina [3,4], and subsequent progressive visual loss. *MYOCILIN*  (*MYOC*; OMIM 601652) was the first gene identified in glaucoma [5]. Mutations in this gene are mainly confined to exon 3, which encodes the olfactomedin-like domain of the protein [6-8]. Heterozygous *MYOC* mutations segregate with the disease in a subset of families with autosomal dominant juvenile-onset, and are present in 3-5% of patients with adult-onset POAG. *MYOC* encodes a 55-57 kDa extracellular glycoprotein of an unknown function that forms homo-oligomers of more than 116 kDa [9-11]. Myocilin shows a modular structure consisting of three domains: 1) the NH_2_-terminal leucine zipper-like region; 2) a central putative linker domain; and 3) the COOH-terminal olfactomedin-like domain. These domains are encoded by exons 1, 2, and 3, respectively. This protein is relatively abundant in the ciliary body, iris, retina, TM [12,13], and in the AH [14]. It is proteolytically cleaved between amino acids Arg226-Ile227 by calpain II in the lumen of the ER [9,15]. A COOH-terminal proteolytic fragment resulting from cleavage between amino acids Glu214-Leu215 has also been reported in HEBNA 293 cells [16]. The processed COOH-terminal domain is secreted into the culture medium, while the NH_2_-terminal fragment mainly remains intracellularly retained [9,15]. It has been suggested that this processing could regulate the interaction of myocilin with other proteins [15].

The mechanism by which mutant myocilin causes the glaucoma phenotype remains elusive. Over recent years however, some hypotheses have been formulated to explain the pathogenicity of *MYOC* mutations. Biochemical and cell biological studies have provided evidence of a gain-of-function disease model [17]. *MYOC* disease-causing mutations produce misfolded polypeptides [18-20] which show reduced secretion both in cells in culture [9,19,21] and in transgenic mice [22-24]. Non-secreted mutant myocilin could compromise the proteosomal function, leading to cell death [20,25,26]. In addition, it has been reported that wild-type/mutant heteromeric aggregates inhibit the secretion of the wild-type protein in cells in culture [11,19,20]. However, the effect of wild-type myocilin on secretion of the mutant protein has not been investigated. Likewise, it has previously been reported that *MYOC* mutations reduce the proteolytic processing of myocilin [9], but whether heterozygosis affects the proteolytic processing of myocilin has not been analyzed.

In the present study, we investigate the pathogenic mechanism by which heterozygous mutations in *MYOC* cause glaucoma. We found that co-expression of wild-type and each of 5 different mutant myocilins, lead to a significant increase in the secretion of the mutant myocilins, along with a reduction of the extracellular wild-type proteolytically unprocessed and processed protein, probably by a dominant negative mechanism mediated by heteroaggregation. We speculate that the extracellular presence of mutant myocilin could be a key element in the molecular mechanisms that lead to glaucoma.

## METHODS

### Myocilin constructs

The cDNAs encoding wild-type and glaucoma-causing myocilin mutants were cloned into the EcoRI-BamHI sites of the pcDNA3.1-myc-His [9] and pcDNA3.1-HA-His mammalian expression vectors, used as previously described [9].

### Cell transfections

The human embryonic kidney 293T (HEK-293T) cell line was bought from the ATCC (American type Culture Collection, Manassas, VA), and it was maintained in Dulbecco’s modified Eagle’s medium (DMEM) supplemented with 10% fetal bovine serum (FBS) and antibiotics (Normocin; Invivogen, Tolousse, France) at 37 °C, in a fully humidified 5% CO_2_ atmosphere. Transient plasmid transfections were carried out with 400 ng of total DNA (200 ng of each cDNA construct in co-transfection assays, and 200 ng of the cDNA construct, plus 200 ng of the non-recombinant pcDNA3.1-myc-His vector in single transfection assays) using the Superfect Transfection Reagent (Quiagen, Valencia, CA), as described [15]. The efficiency of transfections was estimated in cells transiently transfected with a cDNA construct encoding GFP by counting the number of GFP-positive cells in a total of 10^3^ cells in four randomly selected areas per dish. The cells were cultured for either 24 or 96 h after transfection to control the degree of myocilin processing [15].

### Western blotting and antibodies

Analytical 10% polyacrylamide gel electrophoresis in the presence of SDS was performed using the Mini-PROTEAN III gel electrophoresis system (Bio-Rad, Hercules, CA). For western blot analysis, aliquots of culture medium and cell lysates were treated with loading buffer containing β-mercaptoethanol, boiled for 5 min, and fractionated by SDS-PAGE. For nonreducing SDS-PAGE, samples were treated with loading buffer without β-mercaptoethanol, without boiling, and were loaded onto either 8% polyacrylamide gels or 4-15% polyacrylamide gradient gels (Ready Gel, Bio-Rad). Samples were normalized for protein content using the Bradford assay. Alpha-tubulin was detected by western blot using a mouse monoclonal alpha-tubulin antibody (Sigma, St Louis, MO) diluted at 1:5000 as a loading control in cell lysates. Gels were subsequently transferred onto Hybond ECL nitrocellulose membranes (Amersham, Uppsala, Sweden) for immunodetection. Commercial mouse monoclonal anti-myc and anti-HA antibodies (Santa Cruz, Santa Cruz, CA) were used as primary antibodies, diluted at 1:500. A horseradish peroxidase-conjugated antibody against mouse IgG (Pierce Biotechnology, Rockford, IL) was diluted at 1:1000. Chemiluminiscence was performed with Supersignal Dura Western Blot reagents (Pierce). Densitometry for protein band quantitation was performed on scanned films using Quantity One 4.1 analysis software (BioRad) on triplicated independent experiments. The percentage of unprocessed myocilin in either the culture medium or the cellular fraction was calculated as: (extracellular myocilin/total myocilin) x 100 or (intracellular myocilin/total myocilin) x 100, respectively. Total myocilin in each experiment was calculated as the sum of all the myocilin signals in both the culture medium and the cellular fraction. Data were statistically treated by SigmaStat 2.0 software (SPSS Science, Chicago, IL).

## RESULTS

### Analysis of mutant myocilins co-expressed with wild-type myocilin

**Figure 1:**
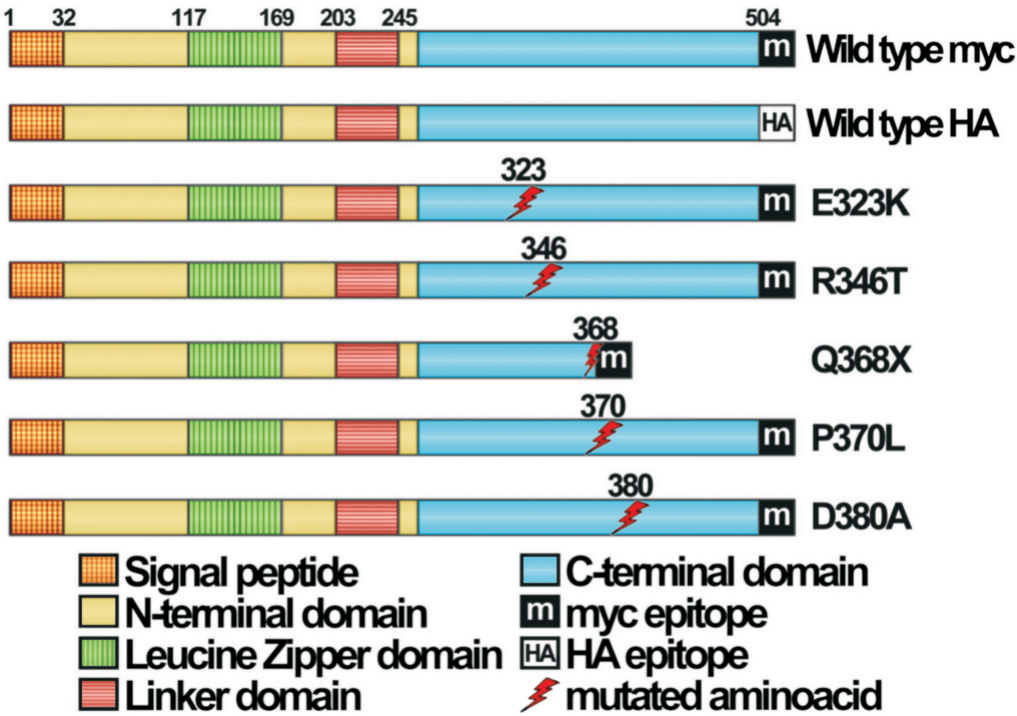
Scheme of wild-type and mutant myocilin cDNA constructs used in this study. The localization of different domains and epitopes of myocilin is indicated by rectangles filled with different patterns. Numbers correspond to positions in the myocilin amino acid sequence.

**Figure 2:**
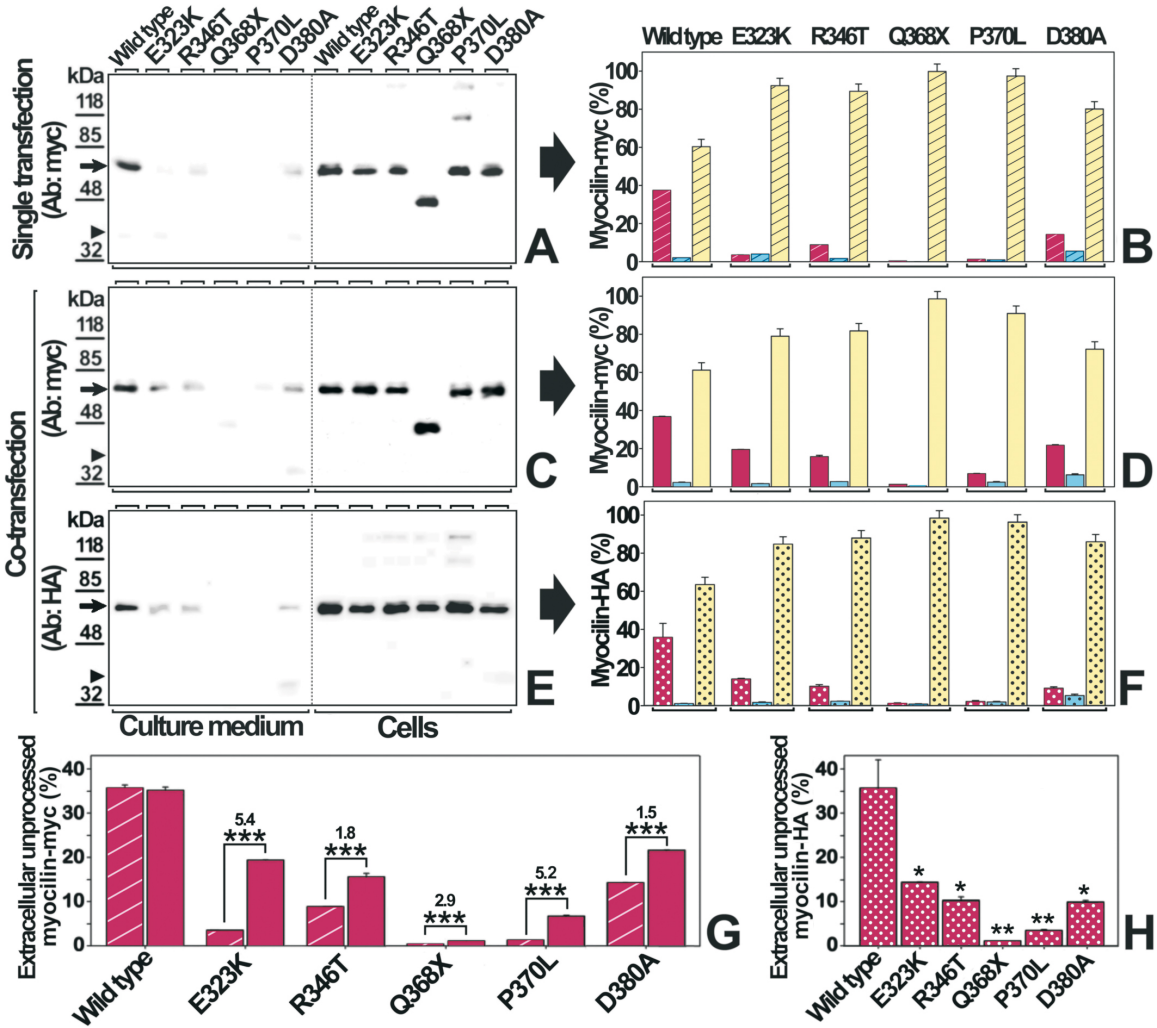
Wild-type/mutant myocilin co-expression significantly increases the amount of extracellular mutant myocilin compared to single mutant expression. HEK-293T cells were transiently transfected with 200 ng of cDNA constructs encoding 5 different myocilin-myc mutants or wild-type myocilin-myc (**A**). To model the heterozygous expression of myocilin mutants, cells were co-transfected with 200 ng of cDNAs encoding each mutant myocilin-myc and 200 ng of a cDNA construct encoding wild-type myocilin-HA. Twenty-four hours after transfection, the recombinant mutant-myc (**C**) and wild-type-HA myocilins (**E**) in both the culture medium and cells were analyzed by 10% polyacrylamide SDS-PAGE and western blot using either an anti-myc or an anti-HA monoclonal antibody, respectively. Arrows and arrowheads indicate the position of full-length myocilin (55 kDa), and the 35 kDa processed olfactomedin-containing fragment, respectively. Molecular weight markers are shown on the left. **B***,* **D** and **F**: Densitometric quantitation of full-length myocilin and its processed olfactomedin-containing fragment, detected in **A** (bars with oblique lines)*,* **C** (solid bars), and **E** (dotted bars). Red bars represent the percentage of extracellular unprocessed full-length or truncated Q368X myocilin (extracellular myocilin/total myocilin), blue bars correspond to the extracellular olfactomedin-containing fragment (extracellular 35 kDa myocilin/total myocilin), and yellow bars represent intracellular full-length myocilin or the truncated Q368X form (intracellular myocilin/total myocilin), respectively. Total myocilin in each experiment was calculated as the sum of all the myocilin signals in both the culture medium and the cellular fraction. Error bars represent the S.E. of triplicate experiments. To facilitate the comparison of the percentage of extracellular unprocessed full-length or truncated Q368X myocilin, the corresponding bars form panels **B** and **D** (**G**) or panel **F** (**H**) are represented together. Numbers above bars in panel **G** indicate the fold-increase of full-length myocilin in cotransfections versus single transfections. Statistical significance as compared with full-length wild-type myocilin, was calculated using the Student’s test. The asterisk indicates a  p<0.05, the double asterisk indicates a  p<0.01 and the triple asterisk indicates a p<0.001.

Most *MYOC* mutations identified today in adult-onset and autosomal dominant juvenile-onset POAG patients are present in the heterozygous state. To study the mechanism by which these mutations lead to glaucoma, we have used a cellular model of heterozygosis. The heterozygous state was simulated by transiently co-expressing in HEK-293T cells, wild-type-HA myocilin, and each of 4 different missense (E323K, R346T, P370L, and D380A) or 1 nonsense (Q368X) mutant myocilins found in glaucoma patients. Mutant proteins were tagged at their COOH-terminal end with the myc-epitope (Figure 1). Three of the missense mutations were selected because they were considered representative of autosomal dominant glaucoma (E323K, P370L, and D380A) [27-29]. The fourth missense mutation (R346T), identified in a Spanish patient with POAG [30] was considered as an example of a myocilin variant involved in non-Mendelian glaucoma, whereas the nonsense mutation Q368X has been found in both autosomic dominant juvenile-onset glaucoma and non-Mendelian cases of glaucoma [5].The HEK-293T cell line was chosen because is well characterized, allows easy expression of recombinant proteins and has been previously used as a cellular model to study different recombinant human proteins including myocilin [9,25,31,32]. The experiments were replicated in COS-1 cells as well as in a cell line derived from the human ciliary muscle (hCM) [9] (data not shown). Initially as a control, we performed a single transfection of HEK-293T cells with the cDNAs encoding wild-type myocilin and each of the 5 pathogenic mutations. By western blot we analyzed the distribution of the mutant proteins in both the culture medium and the cellular fraction 24 h after transfection (Figure 2A). We observed that while 62% of total wild-type myocilin was present intracellularly, more than 80% of each mutant myocilin accumulated intracellularly. Interestingly enough, small amounts of mutant myocilins (ranging from 0.4 to 14.3% of total mutant protein for Q368X and D380A mutations, respectively) were present in the culture medium, mainly as full-length molecules (Figure 2A,B). Next, we studied the effect of wild-type myocilin on the secretion of mutant myocilins. When the cDNAs encoding each mutant myocilin were transiently co-transfected with equal amounts of the cDNA encoding the wild-type protein, the presence of extracellular mutant myocilins increased significantly (Figure 2C,D; red bars). The percentage of missense myocilins in the culture medium ranged from about 7 to 22% for mutants P370L and D380A, respectively. In contrast, the extracellular level of truncated myocilin Q368X was very low (<1.5%), even in co-expression with wild-type myocilin. The fold increase of extracellular full-length mutant myocilins in heterozygosis ranged from 1.5 to 5.4, for mutants D380A and E323K, respectively (Figure 2G).

In the same experiments we also analyzed how mutant myocilins affected secretion of the wild-type protein (Figure 2E,F). The percentage of wild-type myocilin in the culture medium fell significantly (p<0.05) from 35% (control single transfection) to about 9-14% when it was co-expressed with mutants E323K, R346T, or D380A (Figure 2H). The co-expression with mutants Q368X and P370L resulted in a remarkable reduction of the extracellular wild-type protein to approximately 1% (36 fold) and 3.4% (10.5 fold), respectively (Figure 2H).

**Figure 3:**
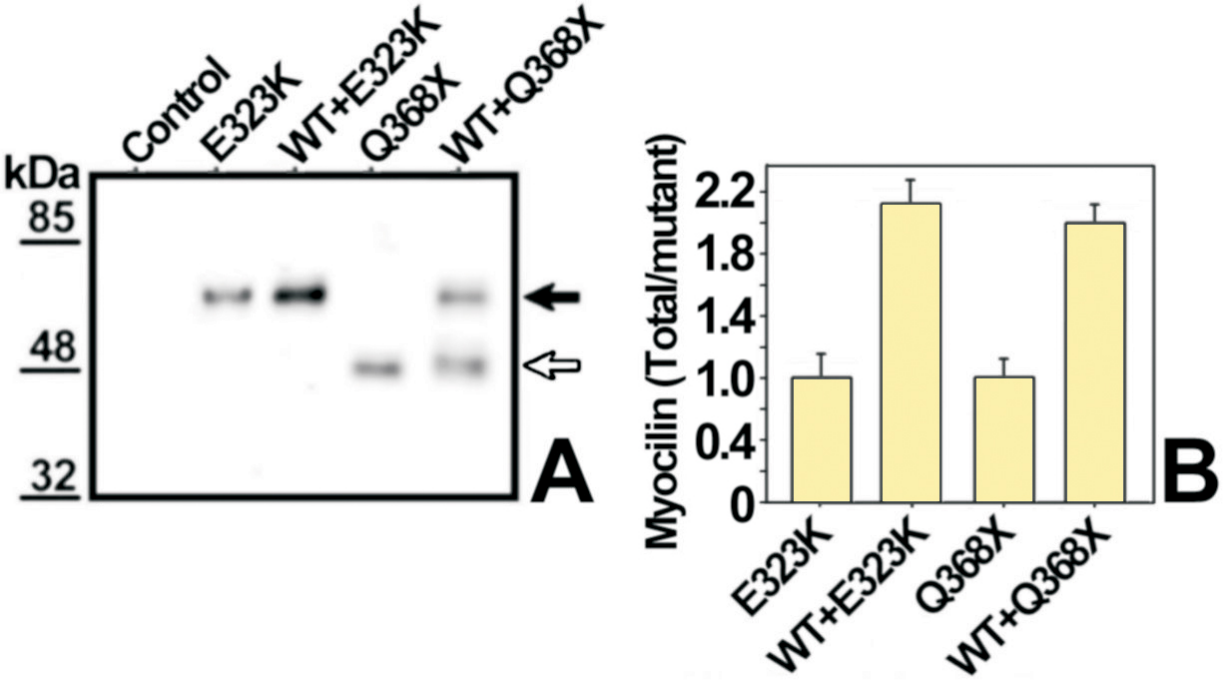
Co-expression of wild-type and mutant myocilin does not alter myocilin levels. **A**: HEK-293T cells were transiently transfected with 200 ng of cDNAs constructs encoding either mutant myocilins E323K or Q368X. These cells were also co-transfected with 200 ng of a cDNA construct encoding wild-type myocilin-HA and 200 ng of either cDNA encoding mutant myocilin-myc E323K or Q368X. Ninety-six hours after transfection, total recombinant myocilin in cell lysates were analyzed by 10% polyacrylamide SDS-PAGE and western blot using the anti-myocilin polyclonal antibody R14T. The black and white arrows indicate the position of full-length myocilin and truncated Q368X myocilin, respectively. Molecular weight markers are shown on the left. **B**: densitometric quantitation of myocilin detected in **A**. Error bars represent the S.E. of triplicate experiments.

The proportion of myc- and HA-tagged wild-type myocilin in the culture medium and cellular fractions was similar in single and co-transfections (Figure 2; wild-type lanes), indicating that co-expression does not affect the distribution of the protein. We confirmed that the expression levels of wild-type and mutant myocilin were similar in co-transfected cells by western blot analysis using an anti-myocilin antibody [33] that recognizes both types of molecules (Figure 3). We did not detect the expression of endogenous myocilin by western blot (Figure 3), which allowed us to rule out any significant interference of the endogenous protein with the different recombinant myocilins expressed in these cells.

### Analysis of the proteolytic processing of myocilin in heterozygosis

**Figure 4:**
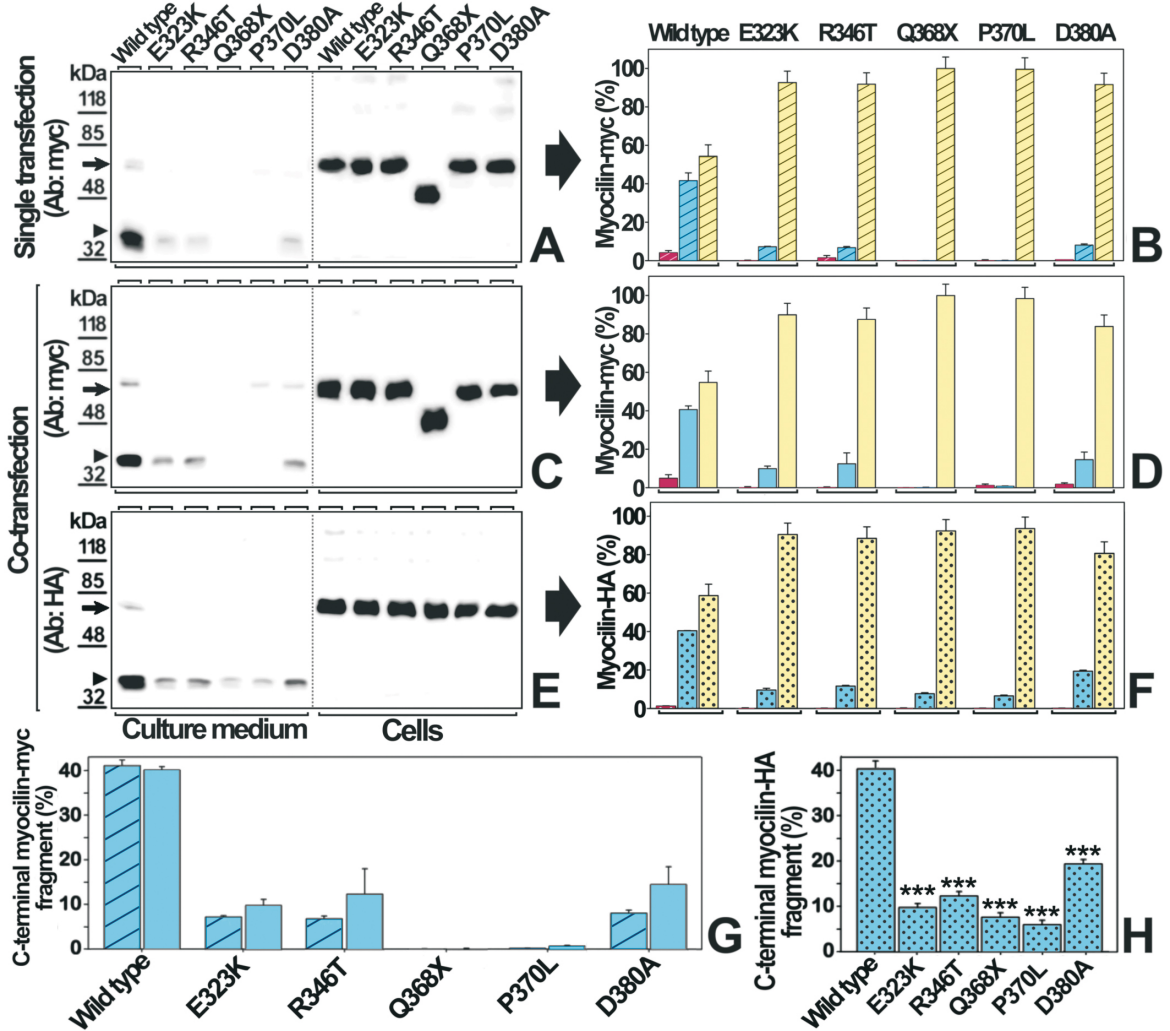
Wild-type/mutant myocilin co-expression reduces significantly the extracellular wild-type olfactomedin cleaved fragment of myocilin. HEK-293T cells were transiently transfected with 200 ng of cDNA constructs encoding the 5 different myocilin-myc mutants or wild-type myocilin-myc (**A**). To model the heterozygous expression of myocilin mutants cells were co-transfected with 200 ng of cDNAs encoding each mutant myocilin-myc and 200 ng of a cDNA construct encoding wild-type myocilin-HA (**C** and **E**). Ninety-six hours after transfection, recombinant mutant and wild-type myocilins-myc in the culture medium and in cell lysates were analyzed by 10% polyacrylamide SDS-PAGE and western blot using either an anti-myc or a anti-HA monoclonal antibody, respectively. Arrows and arrowheads indicate the position of full-length myocilin (55 kDa), and the 35 kDa processed olfactomedin-containing fragment, respectively. Molecular weight markers are shown on the left. **B***,* **D** and **F**: Densitometric quantitation of full-length myocilin and its processed olfactomedin-containing fragment, detected in **A** (bars with oblique lines)*,* **C** (solid bars) and **E** (dotted bars). Red bars represent the percentage of extracellular full-length or truncated Q368X myocilin (extracellular myocilin/total myocilin), blue bars correspond to the extracellular olfactomedin-containing fragment (extracellular 35 kDa myocilin/total myocilin), and yellow bars represent intracellular full-length myocilin or the truncated Q368X form (intracellular myocilin/total myocilin), respectively. The total myocilin in each experiment was calculated as the sum of all the myocilin signals in both the culture medium and the cellular fraction. To facilitate the comparison of the percentage of secreted mutant olfactomedin domain the corresponding bars form panels **B** and **D** (**G**) or panel **F** (**H**) are represented together. Error bars represent the S.E. of triplicate experiments. Statistical significance as compared with the COOH-terminal fragment of wild type myocilin was calculated using the Student’s test. The triple asterisk indicates a p<0.001.

In the next step of the study we analyzed how heterozygous expression affected the processing of both mutant and wild-type myocilin. Transiently transfected cells were cultured for 96 h to increase the extracellular presence of the 35 kDa processed fragment of myocilin [15]. In control single transfections 40% of total wild-type myocilin corresponded to the extracellular processed fragment, 5% to the extracellular full-length protein, and the remaining 55% to the intracellular full-length protein (Figure 4A,B). In contrast, and in accordance with our previous results [9], the percentage of all extracellular mutant COOH-terminal fragments dropped significantly to values varying from about 0.6% (P370L) to 14.5% (D380A) of total mutant myocilin (Figure 4A,B; blue bars). As expected the 35 kDa fragment was not detected in the truncated myocilin Q368X (Figure 4A,B). In co-transfections, all the mutant olfactomedin-containing fragments increased slightly (Figure 4C,D), between 1.3 and 1.8 times compared to single-transfections, although the differences were not significant (p>0.05) (Figure 4G).

The heterozygous expression of wild-type myocilin with each of the 5 mutants for 96 h also produced a remarkable drop in the proportion of the extracellular wild-type 35 kDa processed fragment (Figure 4E,F; blue bars), from 40% of total wild-type myocilin (control) to 20% for the D380A mutant, and to 6%-11% for the other 4 mutants. These values represent a fold reduction ranging from 2 (D380A) to 6.3 (P370L; Figure 4H). Again, and as expected, the COOH-terminal myocilin fragment was not present in the truncated myocilin Q368X (Figure 4C), although the processed COOH-terminal peptide arising from the wild-type counterpart was detected with the HA antibody (Figure 4E). Cell viability and cell lysis at the end of these experiments were determined by the MTT and LDH assays, respectively. No significant effects on these two parameters were observed (data not shown).

### Aggregation of mutant myocilins co-expressed with wild-type myocilin

**Figure 5:**
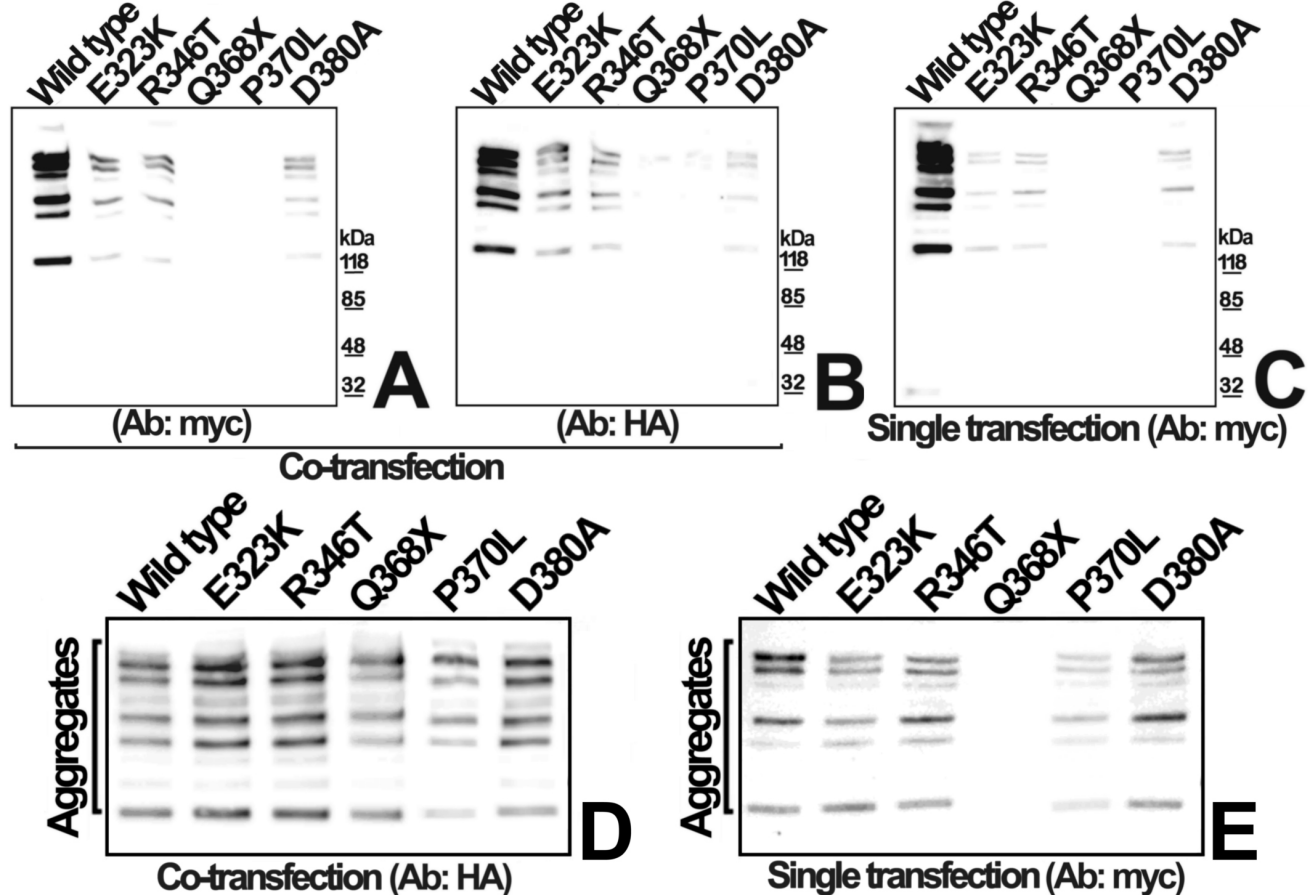
Aggregation of wild-type and mutant myocilins co-expressed for 24 h. HEK-293T cells were transiently transfected with cDNA constructs encoding 5 different myocilin mutants or wild-type myocilin as described in Figure 2. Myocilin aggregates secreted after 24 h of expression were analyzed by nonreducing SDS-PAGE (4-15% gradient polyacrylamide) and were detected by western blot. The culture media from co-transfections were analyzed using either an anti-myc antibody to detect mutant myocilin (**A**), or an anti-HA antibody to detect wild-type myocilin (**B**). Control single transfections were analyzed using an anti-myc monoclonal antibody (**C**). Molecular weight markers are shown on the right of panels **A**, **B** and **C**. To facilitate the comparison of the molecular size of myocilin aggregates different volumes (1 to 50 μl) of culture medium from panels **B** (**D**) and **C** (**E**) were loaded into gels and analyzed by nonreducing SDS-PAGE (8% polyacrylamide).

We reasoned that if glaucoma mutations increased the molecular size of myocilin aggregates, then they could obstruct AH outflow and hence raise IOP. Therefore, we analyzed by nonreducing SDS-PAGE and western blot whether heterozygous expression of myocilin glaucoma mutants affected myocilin self-aggregation. Culture media from cells expressing myocilin for 24 h showed a regular size pattern of myocilin aggregates, consisting of several bands larger than 118 kDa (Figure 5A-C; wild-type lanes), where the lower band probably corresponds to a myocilin dimer. Aggregates disappeared under reducing conditions (Figure 2A), showing that they are maintained by disulphide bonds in accordance with previous reports [10,34,35]. Extracellular myocilin aggregates resulting from transient co-transfection of HEK-293T cells with equal amounts (200 ng) of cDNA constructs encoding wild-type and each of the 4 missense myocilin mutants showed the same pattern and were recognized by both anti-HA and anti-myc antibodies, indicating that they correspond to wild-type/mutant myocilin heteroaggregates (Figure 5A,B). To facilitate the comparison of the molecular size of myocilin aggregates, sample volumes were corrected by normalizing the total amount of myocilin per sample (Figure 5D,E). In addition, the level of the 4 missense mutant myocilins increased in co-expression with the wild-type protein (Figure 5A versus Figure 5C) agreeing with the results presented in Figure 2. These data indicate that mutant/wild-type myocilin heteroaggregation increases the presence of extracellular mutant myocilin and also show that glaucoma mutations do not increase the molecular size of either myocilin homo- or heteroaggregates (wild-type/mutant). Aggregates resulting from the co-expression of Q368X and wild-type myocilin were only recognized by the anti-HA antibody, indicating that they are composed of only the wild-type protein, and suggesting that the olfactomedin domain could be required for correct formation of intermolecular disulphide bonds.

## DISCUSSION

### Heterozygous expression of myocilin mutants

**Figure 6:**
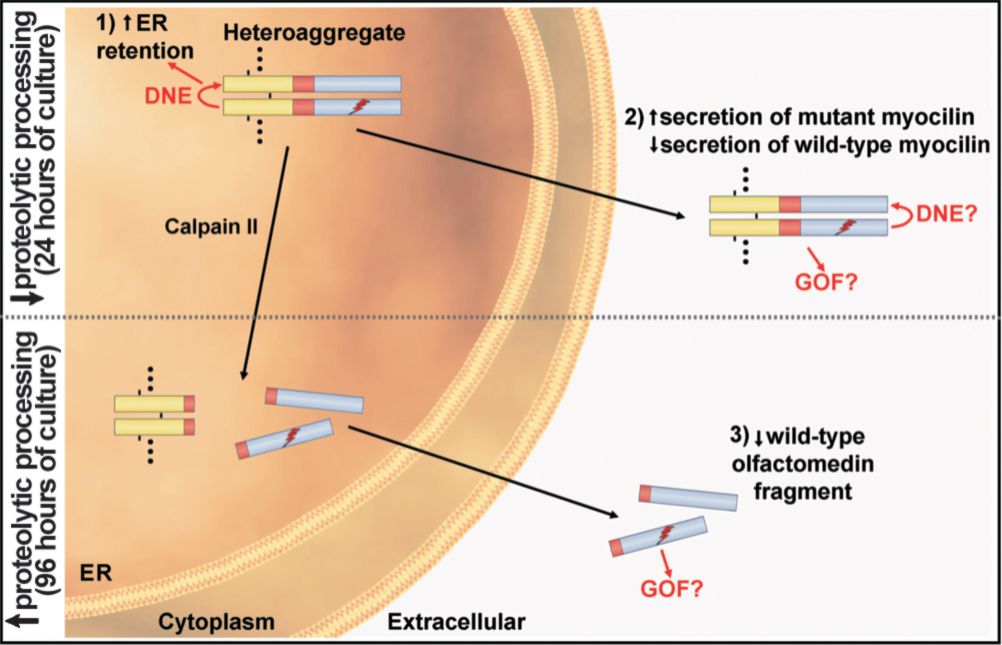
Model of the secretion and proteolytic processing of wild-type/mutant myocilin heteroaggregates. Heteroaggregation takes place in the lumen of the ER. For simplicity the diagram shows a heterodimer but the aggregates are composed of multiple myocilin monomers (indicated by dots in the heteroaggregate) linked by disulphide bonds (short black lines in the heterodimer). According to this model heteroaggregation has three major effects: 1) increases the retention of wild-type myocilin in the ER via a dominant negative effect (DNE); 2) increases secretion of mutant myocilin along with a reduction of extracellular wild-type myocilin, and 3) reduces the amount of extracellular wild-type myocilin (olfactomedin fragment) under conditions known to increase the proteolytic cleavage of myocilin by calpain II such as 96 h of culture [15]. The N-terminal fragment that arises after the cleavage mainly remains in the ER [15]. A possible gain of function (GOF) of mutant myocilin (both full-length and cleaved olfactomedin fragment) that could contribute to glaucoma pathogenesis is also indicated. The leucine zipper (yellow), linker (red), and olfactomedin (blue) domains of myocilin are also indicated in the heterodimer. The ray indicates mutant myocilin. Question marks indicate a hypothetical dominant negative effect and/or gain of function of the mutant protein.

The mechanism by which heterozygous glaucoma mutant myocilins cause either autosomal dominant glaucoma or predispose to non-Mendelian POAG is currently not well understood. Some hypotheses have been proposed over recent years in an attempt to explain myocilin glaucoma pathogenesis. Most of these hypotheses do not consider that *MYOC* mutations involved in glaucoma are usually present in heterozygosis. To gain insight into this issue, we have set up a cellular model of heterozygous expression of wild-type/mutant myocilin in HEK-293T cells. Different groups have consistently shown that pathogenic mutant myocilins transiently expressed in homozygosis accumulate intracellularly in cells in culture, probably as a consequence of protein misfolding [9,18,19,21,24]. Similarly, homozygous transgenic mice expressing mutant myocilin Y423H [22] show reduced secretion of myocilin into the AH, although the animals do not develop glaucoma. Some studies have also shown that certain myocilin mutants, including E323K and D380A, are poorly secreted when expressed alone at 37 °C, and that expressing mutant myocilins at a lower temperature (30 °C), a condition known to facilitate protein folding, enhances their secretion [36]. A few reports have described that mutant myocilin reduces extracellular wild-type myocilin [11,19,20]. However, to the best of our knowledge the effect of wild-type myocilin on mutant myocilin secretion and how mutant myocilin affects the proteolytic processing of wild-type myocilin have not been investigated. In accordance with some of the previous studies, we observed low secretion of the 5 different myocilin mutants transiently expressed in single transfection (homozygosis) at 37 °C. Nevertheless, transient co-expression (heterozygosis) of each of these mutants with the wild-type protein increased the intracellular retention of wild-type myocilin and simultaneously raised the extracellular concentration of mutants (Figure 6; points 1 and 2). Heteroaggregation could explain how mutant and wild type myocilin affect each other’s secretion. The variations observed in the extracellular amount of each myocilin mutant might be due to specific structural defects, which could affect formation of wild-type/mutant heteroaggregates. It is noteworthy that extracellular missense mutant myocilins represented a significant fraction of the total mutant protein expressed in this model of heterozygosis, ranging from 8% to 20% for P370L and D380A mutations, respectively (Figure 2C,D). These findings suggest that the AH and extracellular matrix of the outflow pathway in heterozygous glaucoma patients might contain significant amounts of mutant myocilin, higher than previously thought, forming part of heteroaggregates (wild-type/mutant). The extracellular mutant myocilin in patients could be secreted by different tissues such as the ciliary muscle, ciliary epithelium, and TM cells [33,37,38]. Therefore, it can be also speculated that blocking the secretion of mutant myocilin could be of therapeutic value. It is interesting to note that secretion and processing of myocilin mutations associated with Mendelian cases of glaucoma did not differed significantly from those found in non-Mendelian glaucoma, indicating that other factors should determine the type of inheritance. Our data also suggest that the amount of wild-type myocilin present in the AH could be considerably reduced in heterozygosis (between 2.6 and 10.5 times, for the wild-type myocilin co-expressed with mutants E323K and P370L, respectively). The largest reduction of extracellular wild-type myocilin was produced by the nonsense mutation Q368X. In accordance with our results it has been reported that co-transfection of COS-7 and TM cells with a cDNA encoding wild-type myocilin and increasing amounts of a Q368X cDNA reduced the extracellular wild-type protein [11,19]. Similarly, Joe and co-workers [20] reported that wild-type myocilin-GFP adenovirally co-expressed in TM cells with 4 missense mutant myocilins (G364V, K423E, Y437H, and I477N) mainly accumulated in lysates of human TM cells but was also present in the culture medium. However, another study did not detect wild-type myocilin in the culture medium of TM cells adenovirally co-expressing wild-type and Q368X-GFP myocilin [39]. This discrepancy could be due to different experimental conditions and/or to lower detection sensitivity.

### Molecular mechanisms of myocilin-associated glaucoma

Different mechanisms could contribute to the disease phenotype in myocilin-associated glaucoma. In accordance with our results, and as already mentioned, it has been reported that mutant myocilin inhibits the secretion of the wild-type protein [11,19,20]. These data suggest that wild-type myocilin could be absent in the AH of glaucoma patients carrying *MYOC* mutations. However, reduction or absence of the extracellular normal protein neither explains the apparently normal ocular phenotype in both hemizygous and homozygous *MYOC*-knockout mice [17], nor why mutations such as K423E, which causes glaucoma in heterozygotes, do not produce the disease in homozygotes [40]. From our data we can hypothesize that the presence of extracellular mutant myocilin could be required to produce the disease phenotype, offering an explanation for the lack of glaucoma in K423E homozygous patients. According to this idea, it has been reported that this mutation is not secreted when expressed alone in transiently transfected COS-7 or HTM cells [21], indicating that it could be extracellularly absent in homozygous subjects. However, it might be present extracellularly in heterozygous patients, where it is co-expressed with wild-type myocilin. Likewise, our hypothesis also explains why mutation T377M, which indeed is detected extracellularly in single transient transfections of COS-7 or HTM cells [21], is associated with glaucoma in homozygous POAG patients [41]. Data obtained from some animal models of glaucoma support the role of extracellular mutant myocilin in the pathogenesis of glaucoma. In that sense, it has been reported that non-secretion of mouse mutant myocilin Y423H, which corresponds to human mutation Y437H, is not sufficient to cause glaucoma in *MYOC*-knockin transgenic mice [22]. However, the phenotype of transgenic mouse expressing the same mouse mutant myocilin [42] or human myocilin Y437H [23] in the presence of endogenous wild-type mouse myocilin, show phenotypic features similar to those of POAG in humans (i.e. moderate elevation of IOP, loss of approximately 20% of retinal ganglion cells in the peripheral retina, and axonal degeneration in the optic nerve). The reasons for the phenotypic differences in these mouse models are unclear [43].

Interestingly enough, in this study we have also found for the first time that the extracellular presence of mutant myocilin is associated with a reduction in the amount of extracellular processed wild-type myocilin (olfactomedin domain) by around 2 to 6 times after 96 h in culture. This phenomenon (Figure 6; point 3) could also be due to a dominant negative effect resulting from heteroaggregation and might contribute to the pathogenic mechanism that leads to IOP rising and glaucoma. Dominant negative effects are often caused by mutations in multimeric proteins, and imply that the mutant protein loses its native function and interferes with that of the wild-type protein. According to our previous study [9] we found that the four pathogenic myocilins inhibited the proteolytic processing with varying efficiency. Again we observed that mutation P370L, which produces the most severe glaucoma phenotype, also elicited the most potent endoproteolytic cleavage reduction, in both single and double transfections (Figure 3B,D; blue bars). However, both the actual molecular mechanism by which reduction of the processed myocilin may lead to glaucoma and the physiologic signal(s) that trigger myocilin processing remain to be elucidated. Our hypothesis proposes that the extracellular presence of mutant myocilin is the primary key factor in myocilin-associated glaucoma, which as mentioned previously, is associated with the reduction of both extracellular wild-type myocilin, either as a full-length or proteolytically cleaved protein, and mutant myocilin processing. One can also speculate that a hypothetical gain of function of both extracellular heteroaggregates and mutant olfactomedin-containing fragments (Figure 6) might alter myocilin turnover and/or interactions with other macromolecules, leading to myocilin accumulation over time in the extracellular matrix of tissues of the eye such as the TM and the uveoscleral outflow pathway. Our results do not support an obstruction of the AH outflow by an increase in the molecular size of myocilin aggregates. Further studies are required to determine whether the AH and extracellular matrix from glaucoma patients carrying heterozygous *MYOC* mutations contains myocilin heteroaggregates. According to these ideas it has also been claimed that disease-causing mutations on *MYOC* likely act by a gain of function mechanism [17,20], and/or a dominant negative effect [11,40,44]. Biochemical studies have indicated that the gain of function might be related to several phenomena including: intracellular accumulation in the ER of mutant proteins [9,18,19,32,45], Russell bodies formation and apoptosis [26], cytotoxicity induced by ER stress [20], reduction of its proteolytic processing [9], exposure of a cryptic signaling site that causes mislocalization of the mutant protein to peroxisomes [46], and abnormal interaction(s) between myocilin and ECM and/or cell surface proteins [21]. Our hypothesis does not exclude other proposed mechanisms of myocilin associated glaucoma, instead it complement the current view of glaucoma pathogenesis.

Regarding the genotype-phenotype relationship, we did not find a definitive correlation between the extracellular levels of the 3 missense myocilins and the phenotype, indicating that other unknown factors, such a possible mutation-specific gain of function, could contribute to the phenotype. The Q368X myocilin mutation represents a particular case in glaucoma pathogenesis since it produces a truncate protein which lacks the COOH-terminal half of the olfactomedin-like domain. Whether this difference could explain, at least in part, why Q368X is associated with a milder phenotype and low penetrance remains to be investigated.

In summary, our cellular model of heterozygosis shows that co-expression of wild-type and mutant myocilins increases significantly the presence of extracellular mutant molecules and reduces the amount of either extracellular full-length or processed wild-type myocilin. These data suggests that the abnormal protein could be present through heteroaggregates in the AH and extracellular matrix of the TM and uveoscleral AH outflow of glaucoma patients, playing pivotal roles in the pathogenesis of glaucoma.
